# Prediction of the risk of mortality using risk score in patients with coronary heart disease

**DOI:** 10.18632/oncotarget.13166

**Published:** 2016-11-07

**Authors:** Qian Chen, Ding Ding, Yuan Zhang, Yunou Yang, Qing Li, Xuechen Chen, Gang Hu, Wenhua Ling

**Affiliations:** ^1^ Guangdong Provincial Key Laboratory of Food, Nutrition and Health, Department of Nutrition, School of Public Health, Sun Yat-sen University, Guangdong, China; ^2^ Chronic Disease Epidemiology Laboratory, Pennington Biomedical Research Center, Baton Rouge, Louisiana, USA; ^3^ Department of Cardiology, General Hospital of Guangzhou Military Command of People's Liberation Army, Guangdong, China

**Keywords:** coronary heart disease, risk score, mortality, cohort study

## Abstract

**Background:**

The aim of the study is to develop risk scores with traditional factors for all-cause and cardiovascular mortality among coronary heart disease (CHD) patients.

**Methods and Results:**

We performed a prospective cohort study of 1911 CHD patients aged 40 and older. Cox models were used to estimate the association of traditional factors [sex, age, fasting blood glucose (FBG), high-density lipoprotein cholesterol (HDL-C), low-density lipoprotein cholesterol (LDL-C), blood pressure (BP), and cigarette use] and risk scores with all-cause and cardiovascular mortality. During a mean follow-up of 4.9 years, 232 deaths were identified, 159 of which were cardiovascular-related. Both 4-year and whole follow-up data showed age, sex, HDL-C, LDL-C, and FBG were significantly associated with the risk of mortality, while BP and smoking were not significant predictors in all models. We incorporated age, sex, FBG, HDL-C, and LDL-C to establish risk scores for all-cause and cardiovascular mortality in the 4-year and whole follow-up study. These risk scores were positively associated with the risk of death as quartiles and continuous variables. Assessed by the area under the receiver operating characteristic curves (AUROCs), these risk scores demonstrated strong discriminatory capacity, from 0.744 to 0.763; and the utility of these scores was confirmed with AUROCs from 0.736 to 0.756 (all *P*<0.001) in a validation cohort of 1506 CHD patients with a mean follow-up of 4.7 years.

**Conclusions:**

These simple risk score assessments, including a set of traditional risk factors, might improve the identification of high-risk CHD patients for a more intensive secondary prevention treatment.

## INTRODUCTION

Cardiovascular disease (CVD) is the leading cause of deaths worldwide with more than half from coronary heart disease (CHD) [1]. Although the mortality from heart disease has declined in recent years in the developed countries, the burden of CHD still remains alarmingly high, especially in the developing countries [1-3]. Various risk score systems have been developed to estimate the risk of cardiovascular events (including mortality) within a given time frame among the general population [4-7]. The most common score is from the Framingham Study [5] including age, categories of blood pressure (BP), cigarette use, total cholesterol or low-density lipoprotein cholesterol (LDL-C), high-density lipoprotein cholesterol (HDL-C), and diabetes. Although age, gender, hyperglycemia, dyslipidemia, hypertension and cigarette use are still important for CHD prognosis, convenient and efficient scoring assessments composed of these traditional factors for quantifying the long-term mortality risk among patients with CHD are limited.

Currently, few studies have developed scores or predictions for cardiovascular outcomes among CHD patients [8-10]. Most of these score assessments were developed among acute coronary syndrome (ACS) patients for short-term outcomes prediction, and were all performed in the developed countries. In addition, these scoring systems included many biomarkers, such as angiographic results, electrocardiographic changes or indicators of liver and renal dysfunction, some of which might not be detected for every patient. Practically, a risk stratification tool with routine clinical measurements which would be available for every patient might be simple and easy to understand and calculate by physicians as well as patients. Furthermore, since secondary prevention of CHD is targeted on the risk-reduction clinical treatment and self-management, knowing risk status by patients themselves would have an important impact on supporting and broadening the merits of treatments. The aim of the present study was to develop a simple predictive risk score using traditional factors to estimate the long-term risk of all-cause and cardiovascular mortality among patients with CHD.

## RESULTS

General characteristics of the study population of the Guangdong Coronary Artery Disease Cohort (GCADC) study (as derivation cohort) at baseline were shown in Table 1. We totally studied 1911 CHD patients in the GCADC study; the mean age was 63.6 years, and 65.1% were men. During the mean 4.9 years of follow-up, 232 deaths were identified, 159 of whom were due to cardiovascular causes. Compared with patients who survived during the follow-up, non-survivors were more likely to be older, had lower diastolic blood pressure (DBP) and higher fasting blood glucose (FBG), and had a high proportion of males, less education and less leisure-time physical activity. From the validation cohort of 1506 CHD patients using electronic medical records from the same hospitals, 83 deaths were reported during the mean follow-up period of 4.7 years, 46 of them were cardiovascular-related.

**Table 1:** Baseline characteristics by mortality status among patients with coronary heart disease of the derivation cohort

Characteristic	All patients	Survivors	Non-survivors	*P* for difference
N	1911	1679	232	
Gender (male, %)	65.1	64.9	67.2	0.003
Age at baseline (yrs)	63.6	62.5 (0.26)	71.7 (0.71)	<0.001
Body mass index (kg/m^2^)	23.9	23.9 (0.08)	23.6 (0.22)	0.26
Systolic blood pressure (mm Hg)	134	134 (0.56)	132 (1.54)	0.17
Diastolic blood pressure (mm Hg)	76.5	76.8 (0.31)	74.8 (0.87)	0.036
Fasting blood glucose (mmol/L)	6.47	6.40 (0.07)	7.04 (0.18)	0.001
High-density lipoprotein cholesterol (mmol/L)	41.9	1.09 (0.01)	1.05 (0.02)	0.08
Low-density lipoprotein cholesterol (mmol/L)	115	2.96 (0.02)	3.03 (0.07)	0.30
Triglycerides (mmol/L)	1.85	1.86 (0.03)	1.72 (0.09)	0.14
Total cholesterol (mmol/L)	4.68	4.68 (0.03)	4.72 (0.07)	0.62
Married (%)	90.8	92.2	81.4	0.005
Years of education (%)				0.005
≤9	59.6	58.6	67.5	
10-12	22.1	21.9	23.3	
≥13	18.4	19.5	9.2	
Smoking (%)				0.67
Never	60.4	59.7	65.5	
Past	8.8	8.5	11.6	
Current	30.7	31.8	22.8	
Alcohol drinking (%)				0.71
Never	78.3	77.9	81.2	
Past	6.7	6.7	6.6	
Current	15.1	15.5	12.2	
Leisure-time physical activity (%)				<0.001
None	33.5	31.9	50.0	
≤30 minutes/day	21.6	21.3	24.6	
>30 minutes/day	44.9	46.8	25.4	
Type of coronary heart disease (%)				0.60
Acute coronary syndrome	59.8	59.8	59.5	
Chronic coronary heart disease	40.2	40.2	40.5	
Family history of coronary heart disease (%)	8.7	9.0	6.5	0.68
Use of medication before admission (%)				
Antihypertensive drugs	48.9	47.9	55.7	0.88
Anti-diabetic drugs	16.5	15.7	22.2	0.10
Lipid-lowering drugs	12.1	12.3	10.9	0.60
Anti-platelet drugs	19.7	18.8	25.7	0.13

Abbreviations: Data are mean (standard error) or percentage; all variables are adjusted for age and gender, except for age (adjusted for gender only) and gender (adjusted for age only).

The associations of different levels of selected risk factors at baseline with the risks of all-cause and cardiovascular mortality were shown in Table 2. After multivariate adjustment (alcohol consumption, marriage, family history of CHD, types of CHD, leisure-time physical activity, and BMI), gender, age, FBG, and HDL-C were significantly predictive for all-cause and cardiovascular mortality over 4-year and whole follow-up periods, whereas LDL-C was only predictive for cardiovascular mortality in the whole follow-up period. BP and smoking were not associated with an increased risk of mortality in either 4-year or whole period of follow-up. Systolic blood pressure (SBP) was positively correlated with LDL-C and HDL-C, and DBP was positively correlated with LDL-C and negatively correlated with FBG (all r<0.1, *P*<0.05) (data no shown). Risk scores were given for different sexes, different levels of age, FBG, HDL-C and LDL-C (Table 3). BP and smoking have not been included in the final risk scores because they were not significant predictors in all multivariate models.

**Table 2:** Hazard ratios for all-cause and cardiovascular mortality according to major risk factors among patients with coronary heart disease of the derivation cohort

Variable	No. of CHD patients	4-year follow-up				Whole period of follow-up			
		No. of deaths		Hazard ratios (95% CI) ^a^		No. of deaths		Hazard ratios (95% CI)^a^	
		Total	CVD	All-cause mortality	CVD mortality	Total	CVD	All-cause mortality	CVD mortality
Gender									
Female	666	53	34	1.00	1.00	76	46	1.00	1.00
Male	1245	127	95	1.92 (1.33-2.77)	2.32 (1.49-3.62)	156	113	1.58 (1.15-2.17)	1.95 (1.31-2.89)
Age, years									
<60	682	18	12	1.00	1.00	25	16	1.00	1.00
60-69	569	41	35	3.52 (2.00-6.18)	4.74 (2.43-9.26)	56	44	3.33 (2.06-5.39)	4.33 (2.41-7.77)
≥70	660	121	82	8.20 (4.89-13.7)	8.48 (4.50-16.0)	151	99	7.28 (4.67-11.3)	7.63 (4.38-13.3)
*P* for difference				<0.001	<0.001			<0.001	<0.001
Fasting blood glucose, mmol/L									
<5.6	796	52	31	1.00	1.00	67	37	1.00	1.00
5.6-6.9	381	41	29	1.55 (1.02-2.35)	1.86 (1.11-3.11)	51	33	1.64 (1.13-2.38)	1.89 (1.17-3.04)
≥7.0 or known diabetes	734	87	69	1.75 (1.23-2.48)	2.31 (1.50-3.57)	114	89	1.87 (1.37-2.55)	2.59 (1.75-3.84)
*P* for difference				0.007	0.001			<0.001	<0.001
HDL cholesterol, mmol/L									
<1.03	896	101	77	1.63 (1.17-2.28)	1.94 (1.29-2.92)	125	93	1.53 (1.15-2.05)	1.93 (1.35-2.78)
1.03-1.54	901	64	40	1.00	1.00	89	52	1.00	1.00
≥1.55	114	15	12	2.11 (1.18-3.75)	2.92 (1.50-5.68)	18	14	1.67 (0.99-2.80)	2.42 (1.32-4.44)
*P* for difference				0.003	0.001			0.008	<0.001
LDL cholesterol, mmol/L									
<2.59	709	76	51	1.00	1.00	93	59	1.00	1.00
2.59-4.14	981	83	60	0.95 (0.69-1.30)	1.02 (0.70-1.50)	108	75	0.99 (0.75-1.31)	1.10 (0.78-1.56)
≥4.15	221	21	18	1.29 (0.78-2.12)	1.78 (1.01-3.13)	31	25	1.54 (1.01-2.36)	2.14 (1.31-3.50)
*P* for difference				0.48	0.10			0.086	0.007
Blood pressure, mm Hg									
<120/80	443	38	28	1.00	1.00	50	34	1.00	1.00
≥120-139/80-89	717	61	42	1.04 (0.69-1.57)	1.01 (0.62-1.65)	81	54	1.01 (0.70-1.45)	1.06 (0.68-1.64)
≥140/90	751	81	59	1.05 (0.70-1.57)	1.06 (0.66-1.69)	101	71	0.95 (0.67-1.36)	1.03 (0.67-1.58)
*P* for difference				0.97	0.97			0.92	0.97
Smoking status									
Never	1155	117	83	1.00	1.00	152	102	1.00	1.00
Past	169	18	14	0.84 (0.50-1.42)	0.87 (0.48-1.58)	27	18	1.01 (0.65-1.57)	0.92 (0.54-1.57)
Current	587	45	32	0.80 (0.54-1.18)	0.73 (0.46-1.17)	53	39	0.76 (0.53-1.09)	0.74 (0.49-1.13)
*P* for difference				0.49	0.42			0.28	0.37

Abbreviations: CHD, coronary heart disease; CI, confidence interval; CVD, cardiovascular disease; HDL, high-density lipoprotein; LDL, low-density lipoprotein.

a Adjusted for alcohol consumption, marriage, family history of coronary heart disease, type of coronary heart disease, leisure-time physical activity, and body mass index.

**Table 3:** Risk score coefficients for all-cause mortality and cardiovascular mortality over 4 years and whole follow-up

Year of follow-up	4-year of follow-up		Whole period of follow-up	
	All-cause mortality	CVD mortality	All-cause mortality	CVD mortality
Gender				
Female	0	0	0	0
Male	7	8	5	7
Age, years				
<60	0	0	0	0
60-69	13	16	12	15
≥70	21	21	20	20
Fasting blood glucose, mmol/L				
<5.6	0	0	0	0
5.6-6.9	4	6	5	6
≥7.0 or known diabetes	6	8	6	10
HDL cholesterol, mmol/L				
<1.03	5	7	4	7
1.03-1.54	0	0	0	0
≥1.55	7	11	5	9
LDL cholesterol, mmol/L				
<2.59	0	0	0	0
2.59-4.14	0	0	0	1
≥4.15	3	6	4	8

Abbreviations: CVD, cardiovascular disease; HDL, high-density lipoprotein; LDL, low-density lipoprotein.

When we stratified risk scores into quartiles, the risk scores during both 4-year and the whole period of follow-up showed a graded increased association with the risks of all-cause and cardiovascular mortality (all *P*>0.001) (Table 4). Compared with patients in the lowest risk group (quartile 1 of risk score), the multivariable-adjusted hazard ratios (HRs) for all-cause and cardiovascular mortality among patients with the highest risk (quartile 4 of risk score) were 10.6 [95% confidence interval (CI): 5.66-20.0] and 15.1 (95% CI: 6.53-35.0) over 4-year follow-up; 8.86 (95% CI: 5.15-15.3) and 14.5 (95% CI: 6.68-31.6) over the whole follow-up, respectively. Each 1 value increase in risk score contributed to appropriately 10% increased risk of all-cause mortality (HR 1.10, 95% CI: 1.08-1.13 for 4-year follow-up and HR 1.11, 95% CI: 1.09-1.13 for whole period of follow-up) and cardiovascular mortality (HR 1.11, 95% CI: 1.08-1.13 for 4-year follow-up and HR 1.10, 95% CI: 1.08-1.12 for whole period of follow-up). The discrimination for risk scores was shown in Table 5. Assessed by the area under the receiver operating characteristic curve (AUROC), these four risk score models for risks of all-cause and cardiovascular mortality over 4-year and whole period of follow-up demonstrated the discriminatory capacity from 0.744 to 0.763 (all *P*<0.001) for the derivation cohort and 0.736 to 0.756 (all *P*<0.001) for the validation cohort, respectively.

**Table 4:** Hazard ratios for all-cause and cardiovascular mortality according to risk score quartiles among patients with coronary heart disease of the derivation cohort over 4 years and the whole follow-up

Model	Quartiles of risk score				*P* for trend	Each 1 score increase
	Quartile 1	Quartile 2	Quartile 3	Quartile 4		
4-year of follow-up						
All-cause mortality						
Range of scores	0-13	14-21	22-29	30-42		
No. of CHD patients	510	443	482	476		
No. of deaths	11	14	57	98		
Multiple adjusted hazard ratios^a^	1.00	1.51 (0.69-3.34)	6.18 (3.23-11.8)	10.6 (5.66-20.0)	<0.001	1.10 (1.08-1.13)
Cardiovascular mortality						
Range of scores	0-16	17-27	28-35	36-50		
No. of CHD patients	503	471	475	462		
No. of deaths	6	9	42	72		
Multiple adjusted hazard ratios^a^	1.00	1.63 (0.58-4.58)	7.94 (3.36-18.8)	15.1(6.53-35.0)	<0.001	1.11 (1.08-1.13)
Whole period of follow-up						
All-cause mortality						
Range of scores	0-11	12-20	21-26	27-39		
No. of CHD patients	465	484	469	493		
No. of deaths	15	24	71	122		
Multiple adjusted hazard ratios^a^	1.00	1.64 (0.86-3.14)	5.44 (3.11-9.53)	8.86 (5.15-15.3)	<0.001	1.11 (1.09-1.13)
Cardiovascular mortality						
Range of scores	0-17	18-26	27-34	35-52		
No. of CHD patients	448	484	509	470		
No. of deaths	7	12	51	89		
Multiple adjusted hazard ratios^a^	1.00	1.71 (0.67-4.35)	7.25 (3.28-16.0)	14.5 (6.68-31.6)	<0.001	1.10 (1.08-1.12)

Abbreviations: CHD, coronary heart disease.

a Adjusted for smoking, alcohol consumption, marriage, family history of coronary heart disease, types of coronary heart disease, leisure-time physical activity, blood pressure categories, and body mass index.

**Table 5:** Discrimination measured by AUROC for risk score over 4 years and whole period of follow-up

Model	AUROC	*P* value
Derivation cohort		
4-year of follow-up		
Risk score for all-cause mortality	0.754 (0.72-0.79)	<0.001
Risk score for cardiovascular mortality	0.763 (0.72-0.80)	<0.001
Whole period of follow-up		
Risk score for all-cause mortality	0.744 (0.71-0.78)	<0.001
Risk score for cardiovascular mortality	0.762 (0.73-0.80)	<0.001
Validation cohort		
4-year of follow-up		
Risk score for all-cause mortality	0.756 (0.70-0.82)	<0.001
Risk score for cardiovascular mortality	0.736 (0.67-0.80)	<0.001
Whole period of follow-up		
Risk score for all-cause mortality	0.736 (0.68-0.79)	<0.001
Risk score for cardiovascular mortality	0.743 (0.69-0.80)	<0.001

Abbreviations: AUROC, area under receiver operating characteristic curve.

## DISCUSSION

We developed a simple risk score system (including sex, age, FBG, HDL-C, and LDL-C) for estimating prognosis of all-cause and CVD mortality among Chinese patients with CHD over a 4-year and a whole period of follow-up.

In the present study, we observed that men with CHD were at higher risk of mortality than women with CHD, which was the same as previous studies [4, 8]. As expected, age was always found to be the strongest predictor of the risk of mortality among patients with CHD. For other traditional factors studied, both impaired fasting glucose (FBG: 5.6-6.9 mmol/L) and diabetes were significantly associated with increased risks of mortality among CHD patients. We also found that the hazard ratios of mortality associated with FBG increased slightly with the increased length of follow-up, which indicated that FBG levels might have a significant long-term impact on CHD prognosis. The U-shape association of HDL-C with the risk of mortality was consistent with other studies [11, 12]. Under particular conditions, HDL would lose its protective functions (antioxidant, anti-inflammation, anti-apoptotic, ameliorate endothelial dysfunction) and gain dysfunction, which might contribute to inflammatory processes that promote atherosclerosis in CHD patients [13]. It suggests that controlling HDL-C at a reasonable range is necessary. Since LDL-C and HDL-C would be always detected as a pair of lipoprotein fractions in routine clinical examinations, we selected LDL-C, which was positively associated with the risk of mortality among CHD patients, instead of total cholesterol in the present study. High BP and cigarette use have been thought to be strong predictors for mortality among the general population. However, in the present study, we did not find any association of BP and cigarette use with the risk of mortality among patients with CHD. The lack of significant association between BP and risk of mortality might be due to high prevalence of using anti-hypertensive medication (48.9%) among patients with CHD. As recommended by the guidelines [14], the tobacco user would be asked to quit smoking and pay more attention to secondary prevention, which partially explained the non-significant results. Taking multiple risk factors into account simultaneously might optimize the ability to estimate the prognosis of CHD patients. To the best of our knowledge, this is the first study that developed risk score assessments in Chinese patients with CHD.

Several previous studies have developed risk prediction scores for CVD risk among CHD patients with an acute setting [9]. TIMI risk score [15] provides a convenient bedside risk score for predicting 30-day mortality in patients with ST-elevation myocardial infarction while PURSUIT risk score [16] predicts 30-day outcomes for patients with ACS but without persistent ST-segment elevation. Granger *et al*. used 11,389 ACS patients enrolled in the Global Registry of Acute Coronary Events (GRACE) to develop the GRACE risk score for predicting mortality, but the median time of death was 4 days after hospital presentation [17]. Although these scoring assessments have been demonstrated significant discriminatory ability for mortality [9], they could not avoid the potential bias caused by premature death or the presence of occult diseases at baseline with the very short-term follow-up, and could not extend to all CHD outpatients.

Marschner et al. [8] developed a long-term risk stratification with 8557 CHD patients in the Long-Term Intervention With Pravastatin in Ischemic Disease (LIPID) study in Australia and New Zealand with an acute cardiac event 3-36 months before inclusion, which had been proposed as one of the first risk assessment scores for both ACS and stable CHD patients. As a strong predictor for prognosis of CHD in our study, LDL-C was not included in LIPID score because of missing data [8]. HDL-C and diabetes status were only divided into two classes in the LIPID risk score. Battes et al. [18] recently developed a cardiovascular risk assessment model in patients with established coronary artery disease, consisting of 12,218 patients in EUROPA database, which also used diabetes status instead of glucose concentrations. In contrast to their results, we found that non-diabetic glucose concentrations (impaired fasting glucose, FBG: 5.6-6.9 mmol/L) and higher HDL-C levels (≥1.55 mmol/L) were associated with an increased risk of mortality, indicating that including FBG and HDL-C as dichotomization variables might be simple to calculate, but it would result in inaccuracy for risk stratification by missing important information.

Another kind of risk scores has been developed in the CHD population for predicting long-term cardiovascular outcomes to stratify CHD patients before therapy like percutaneous coronary intervention (PCI) and coronary artery bypass grafting (CABG) surgery [19-21]. Thus, some complicated variables like severity of illness (number of diseased vessels, ejection fraction, and hemodynamic state, et al.) and the presence of several comorbidities and prior history (cerebrovascular disease, peripheral arterial disease, congestive heart failure, malignant ventricular arrhythmia, chronic obstructive pulmonary disease, diabetes mellitus, and renal failure et al.) would be included in the risk stratification. Complicated procedures might result in seldom use in the clinic. Unlike these previous risk scores for CHD patients, our risk scores only integrated available traditional measurements which could be detected for every patient routinely and easy to use. Of course, incorporating more clinical results or novel biomarkers would increase the predictive discrimination [22]. But it does not meet our original objective for building a simple and economical assessment; and additional costs for the measurements of unconventional biomarkers might limit the use of risk scores in clinical practice, especially in the developing countries. More importantly, our present risk scores, validated in the external cohort (AUROCs from 0.736-0.756, all *P*<0.001), could also offer strong discriminatory capacity when assessing with AUROC.

There are several limitations in the present study. First, we enrolled participants from hospitals where in-patients may have a more severe disease status, bias may occur. But we recruited both acute and chronic CHD patients, and some of them were electively admitted patients with mild status in order to reduce the bias. Second, although our analyses adjusted for an extensive set of CVD confounding factors, residual confounding due to the measurement error in the assessment of confounding factors or unmeasured factors for all CHD patients cannot be excluded. Third, we only measured the risk factors at baseline and did not further measure them during follow-up. Thus, more studies are warranted to confirm our risk assessments.

In conclusion, we have developed predictive risk scores for all-cause and cardiovascular mortality for patients with CHD. These simple risk scores are based on routinely available clinical information and could be easily calculated. These simple risk scores might be useful, in addition to CHD treatments and the shared decision-making tools between physicians and patients for a more intensive secondary prevention treatment.

## MATERIALS AND METHODS

### Study population

GCADC study was conducted as a derivation cohort. Details of the GCADC study about aims, selection, criteria and ascertainment of CHD have been published previously [23, 24]. Participants who were admitted to the Cardiology Department of three superior specialty hospitals in Guangdong (Guangzhou Military General Hospital, Sun Yat-Sen Memorial Hospital, and First Affiliated Hospital of Sun Yat-Sen University) and diagnosed as coronary artery disease [International Classification of Diseases (ICD)-10 codes I20-I25] according to World Health Organization 1999/2000 guidelines [25, 26] between October 2008 and December 2011were recruited [23]. Briefly, we included 1911 CHD patients of 40 to 85 years old after excluding participants with incomplete data at baseline (n=69). Patients with ACS were defined as the occurrence of any of unstable angina pectoris, ST-segment elevation myocardial infarction, and non-ST-segment elevation myocardial infarction within 3 months. Using the same inclusion and exclusion criteria and diagnosis of CHD as the GCADC study, we additionally recruited 1615 CHD patients via electronic medical records. After excluding 109 patients with incomplete data, we finally enrolled 1506 participants as the external validation cohort. Written informed consent was obtained from each patient of GCADC study; we did not obtain informed consent from participants in the validation cohort because we used anonymized data compiled from electronic medical records. The study plan for the GCADC study and the whole analysis plan were conducted according to the principles expressed in the Declaration of Helsinki and approved by the Sun Yat-Sen University Ethics Committee.

### Measurements

In the GCADC study, each patient's general information of examination date, birth date, gender, address, education level, marriage, leisure-time physical activity, smoking habits, alcohol consumption, dietary intake, family history of diseases, and use of medication before admission was ascertained by a standardized questionnaire and a validated food frequency questionnaire [27] through a face-to-face interview. We classified smoking habits and alcohol consumption into three groups: never, past, or current. Current smoking was defined as regularly at least one cigarette per day and lasting for more than 6 months before the study, and current alcohol drinking was defined as drinking any type of alcoholic beverages at least once a week and lasting for half a year before the study. Family history of CHD was self-reported, including CHD history for all first-degree relatives.

Clinical characteristics of patients from the derivation and validation cohorts were extracted from the electronic record system. At admission, height and weight were measured by trained nurses. Body mass index (BMI) was defined as the weight in kilograms divided by the square of height in meters. Two BP determinations were made after the patients had been sitting at least 5 minutes, and the average was used for the analyses. Blood was drawn at the baseline examination after an overnight fasting. Lipids and FBG were determined by standardized methods using the Hitachi automatic analyzer 7600-020 (Hitachi, Tokyo, Japan).

### Prospective follow-up

Annual follow-up information was obtained from hospital medical records of readmission, telephone contacts with patients or their immediate family members, and death registration at the Guangdong Provincial Center for Disease Control and Prevention. The current surveys were followed to the end of June 2015 or patient's death if the date was prior to June 2015. We used the ICD codes to code the cause of death; the ICD codes I00–I99 were classified as CVD deaths.

### Statistical analyses

Differences in baseline characteristics from the derivation cohort according to death status were tested by the general linear model for continuous variables and logistic regression for categorical variables after adjusting age and gender (except for age and gender). Major traditional risk factors (age, FBG, HDL-C, LDL-C, BP, and cigarette use) have been grouped into three classes: (1) age, <60, 60-69, ≥70 years; (2) FBG, <5.6, 5.6-6.9, ≥7.0 mmol/L and known diabetes (diabetes diagnosed before the baseline examinations); (3) HDL-C, <1.03, 1.03-1.54, ≥1.55 mmol/L; (4) LDL-C, <2.59, 2.59-4.14, ≥4.15 mmol/L; (5) BP, <120/80, 120-139/80-89, ≥140/90 mm Hg; (6) smoking status, never, past, current. Cox proportional hazards models were performed to determine the associations between baseline major risk factors (gender, age, FBG, HDL-C, LDL-C, BP, and cigarette use) and the risk of all-cause and CVD mortality in both 4-year and whole follow-up periods, respectively. All analyses were adjusted for alcohol drinking, marriage, leisure-time physical activity, family history of CHD, type of CHD, and BMI. The selected risk factors were included as categories in the risk score systems to allow for possible non-linear effects of the factors and so that a risk score can be evaluated for a given individual by simply summing scores corresponding with the categories for each factor. We gave a risk score for each category of each factor, except for BP and smoking status because BP and smoking were not significant predictors in all multivariable-adjusted Cox models. These scores were beta coefficients, formed with the Cox model, multiplied by 10 and rounded to the closest integer [4]. The risk score for an individual was obtained by summing the scores for the appropriate level of each of the risk factors. The associations of risk scores for all-cause and cardiovascular mortality over 4-year and whole period of follow-up were estimated by Cox models after adjusting for smoking, alcohol drinking, marriage, leisure-time physical activity, family history of CHD, types of CHD, BP categories, and BMI. Risk scores were included in the Cox model in two ways: as quartiles and as continuous variables. We additionally conducted the Pearson correlation coefficients to assess the correlation between BP and other traditional factors.

The discrimination of capacity of different risk score groups for the derivation cohort and the validation cohort was assessed by the AUROC. Statistical significance was considered to be *P*<0.05. All statistical analyses were performed using PASW for Windows, version 20.0 (IBM SPSS Inc., Chicago, IL).
